# Control of *Streptomyces alfalfae* XY25^*T*^ Over Clubroot Disease and Its Effect on Rhizosphere Microbial Community in Chinese Cabbage Field Trials

**DOI:** 10.3389/fmicb.2021.641556

**Published:** 2021-06-18

**Authors:** Yuanliang Hu, Lu Qiu, Zongjie Zhang, Kai Liu, Xian Xia, Shuanglian Xiong, Shumiao Zhao, Zhuqing Zhao, Yongmei Hu, Yunxiang Liang

**Affiliations:** ^1^Hubei Key Laboratory of Edible Wild Plants Conservation and Utilization, College of Life Sciences, Hubei Normal University, Huangshi, China; ^2^State Key Laboratory of Agricultural Microbiology, College of Life Science and Technology, Huazhong Agricultural University, Wuhan, China; ^3^Hubei Key Laboratory of Soil Environment and Pollution Remediation, College of Resources and Environment, Huazhong Agricultural University, Wuhan, China

**Keywords:** clubroot, quantitative real-time PCR, high-throughput sequencing, *Streptomyces alfalfae*, physicochemical properties

## Abstract

Clubroot caused by *Plasmodiophora brassicae* is one of the most destructive diseases in cruciferous crops. *Streptomyces alfalfae* XY25^*T*^, a biological control agent, exhibited great ability to relieve clubroot disease, regulate rhizosphere bacterial and fungal communities in Chinese cabbage, and promote its growth in greenhouse. Therefore, field experiments were carried out to investigate the effects of *S. alfalfae* XY25^*T*^ on clubroot and rhizosphere microbial community in Chinese cabbage. Results showed that the control efficiency of clubroot by *S. alfalfae* XY25^*T*^ was 69.4%. Applying the agent can alleviate soil acidification; increase the contents of soil organic matter, available nitrogen, available phosphorus, and available potassium; and enhance activities of invertase, urease, catalase, and alkaline phosphatase. During Chinese cabbage growth, bacterial diversity decreased first and then increased, and fungal diversity decreased gradually after inoculation with *S. alfalfae* XY25^*T*^. High-throughput sequencing analysis showed that the main bacterial phyla were Proteobacteria, Bacteroidetes, Acidobacteria, and Planctomycetes, and the major fungal phyla were Ascomycota and Basidiomycota in rhizosphere soil. The dominant bacterial genera were *Flavobacterium*, *Candidatus*, *Pseudomonas*, *Stenotrophomonas*, *Sphingomonas*, *Flavisolibacter*, and *Gemmatimonbacteria* with no significant difference in abundance, and the major fungal genera were *Monographella*, *Aspergillus*, *Hypocreales*, *Chytridiaceae*, *Fusarium*, *Pleosporales*, *Agaricales*, *Mortierella*, and *Pleosporales*. The significant differences were observed among *Pleosporales*, *Basidiomycota*, *Colletotrichum*, two strains attributed to *Agaricales*, and another two unidentified fungi by using *S. alfalfae* XY25^*T*^. Moreover, quantitative real-time PCR results indicated that *P. brassicae* content was significantly decreased after the agent inoculation. In conclusion, *S. alfalfae* XY25^*T*^ can affect rhizosphere microbial communities; therefore, applying the agent is an effective approach to reduce the damage caused by clubroot.

## Introduction

Cruciferous plants are widely planted worldwide and occupy an important position in agricultural production (Botero et al., 2019). Clubroot disease is a highly destructive soil-borne disease caused by the pathogen *Plasmodiophora brassicae* (Dixon, 2014). Cruciferous crops are highly susceptible and are greatly affected by clubroot diseases. The prevention and treatment of cruciferous clubroot disease have always become the focus of research. Rotation, applying chemical agents such as cyclophosphamide, and breeding resistant varieties can inhibit the spore vitality of *P. brassicae* and reduce the occurrence of clubroot disease (Peng et al., 2014; Yang et al., 2020). However, these methods can only alleviate crop clubroot disease, but not completely eliminate it; moreover, the residue of chemical agents will endanger food safety and human health (Lee et al., 2008; Kowata-Dresch and May-De Mio, 2012; Botero et al., 2019; Mehraj et al., 2020). In addition, *P. brassicae* has strong pathogenicity and rapid mutation, and the resistance of crop-resistant varieties is weak (Hirai, 2006; Strelkov et al., 2018). Therefore, these control methods have certain restrictions.

Biological control is an effective control method by applying microbial fertilizers or agents to change the composition of the root soil microbial community and soil acidity and alkalinity, thus affecting the absorption of water, soil organic matter, and other nutrients by crops, and promote the growth of crops (O’Brien, 2017; Rui et al., 2017; Liu et al., 2018). *Streptomyces* is the largest genus of *Actinomycetes*, and the active ingredients produced by this strain play a key role in maintaining the balance of soil microorganisms; therefore, it was widely used as biological control agents (BCAs) for plant diseases (Minuto et al., 2005; Chen et al., 2016). *S. alfalfae* XY25^*T*^ is a BCA identified by She et al. (2016) and it was a Gram-positive, aerobic bacterium with extensive branched substrate mycelia and aerial. Our recent findings found that this strain could improve continuous cropping obstacles and promote crop growth, and it showed good application prospects in the prevention and treatment of clubroot diseases under greenhouse conditions (Liu et al., 2020).

Rhizosphere refers to the soil environment including the root system and its surrounding areas. It is a place where pathogens attack plants and establish parasitic relationships (Kumar et al., 2020). Like other habitats, the rhizosphere contains a large number of interacting microbial populations, and it plays an important role in maintaining plant health (Wei et al., 2019; Daval et al., 2020). Rhizosphere microorganisms prevent or resist the invasion of soil-borne diseases through antagonism, nutrient competition, parasitism, and group sensing (Mendes et al., 2013), and the imbalance of microbial community in rhizosphere soil is an important cause of soil-borne diseases. Improper fertilization, lack of soil nutrients, and accumulation of root exudates lead to changes in the soil microenvironment, resulting in selective adaptation of rhizosphere soil microorganisms, enriching certain specific microbial populations, breaking the soil microecological balance, and eventually causing plant disease (Sudini et al., 2011; Yang et al., 2020).

Cabbage, radish, and other cruciferous vegetables are mainly cultivated in the alpine region in western Hubei Province, which is the largest off-season vegetable production base in Hubei with a planting area of more than 145,400 ha in 2019 (Jiao et al., 2020). Vegetable cultivation is the pillar industry to increase local farmers’ income. However, in recent years, clubroot has spread all over the vegetable area, and the clubroot incidence in Chinese cabbage fields has reached up to more than 60%, which has become the main problem faced by the alpine vegetable industry development in western Hubei (Liu et al., 2020). Our previous pot experiments revealed some obvious inhibiting effects of *S. alfalfae* XY25^*T*^ on clubroots disease. However, there is still lack of evidence of *S. alfalfae* XY25^*T*^ to be used in fields. Therefore, Chinese cabbage field planting experiments in the clubroot high-risk area were conducted to evaluate the control effect of *Streptomyces* on clubroot disease, and quantitative real-time PCR and high-throughput sequencing analysis were performed to reveal the influence of this strain on rhizosphere microorganisms.

## Materials and Methods

### Strain Inoculation and Preparation

*Streptomyces alfalfae* XY25^*T*^ was obtained from State Key Laboratory of Agricultural Microbiology, Huazhong Agricultural University, Wuhan, China, and stored at the Chinese Type Culture Collection Center (CCTCCAA2015019T). The strain was inoculated in liquid Gauze’s synthetic medium at 28°C for 72 h on a rotary shaker (180 rpm). The cells were harvested immediately after centrifugation at 5,000 rpm for 10 min and diluted with sterile distilled water (SDW) for subsequent use.

### Field Experiment and Sampling

The experiment was carried out from August to October 2018. The vegetable growing area of Miaoziling Village, Huoshaoping Township, Changyang County, Yichang City, Hubei Province, China (30.48N, 110.77E), was selected as the experimental site, where the altitude is 1,833 m with average annual temperature of 7.6°C and total rainfall of about 1,500 mm/year. In the selected field, severe clubroot disease had occurred, and it had lots of clubroot pathogens in the soil. The seedlings of Chinese cabbage at the four-leaf stage were transplanted into the field, which was divided into six plots. Three of the plots were randomly assigned to the treatment group (S), and the other three were assigned as the control group (C). An aliquot of 10 ml of *S. alfalfae* XY25^*T*^ (1 × 10^6^ CFU/ml) was inoculated into the root rhizosphere of each plant in the treatment group at 0, 14, and 28 days after transplantation. Meanwhile, the plants in the control group were treated with the same volume of SDW. Before each inoculation of BCA or SDW, the rhizosphere soil samples of plants were collected by root-shaking method (Bakker et al., 2015) at days 0, 14, 28, and 42, respectively. Two plants were randomly selected from each plot for investigation, and the soil samples in each group contained six biological replicates. Each sample was randomly divided into two parts. One part was immediately refrigerated for the determination of physicochemical index and enzyme activity, and the other part was immediately frozen and stored at −80°C for DNA extraction. At the end of the experiment (day 42), all the plants were pulled out, and their root disease incidence and disease index were evaluated according to the previous study (Liu et al., 2018).

### Physicochemical Index and Enzyme Activity Determination

Soil pH was determined with a pH meter after shaking soil–water suspension (1:2.5) for 30 min. The soil samples were air dried, ground, and sieved (<2 mm). The contents of soil organic matter (SOM), available nitrogen (AN), available phosphorus (AP), and available potassium (AK) were measured by potassium dichromate titration, alkaline hydrolysis diffusion, molybdenum–antimony colorimetry, and flame photometer method, respectively (Yang et al., 2017). The 3,5-dinitrosalicylic acid colorimetry (Frankeberger and Johanson, 1983), indophenol blue colorimetry (Huang et al., 2012), disodium phosphate colorimetry, and potassium permanganate titration method (Johnson and Temple, 1964) were used to determine the activity of invertase, urease, alkaline phosphatase, and catalase, respectively.

### DNA Extraction, High-Throughput Sequencing, and Data Analysis

Soil DNA was extracted from the soil samples with E.Z.N.A Soil DNA Kit (Omega, Norcross, Georgia, United States). The quality and quantity of the extracted DNA were checked by 1% agarose gel electrophoresis and a Nanodrop One spectrophotometer (Thermo, Waltham, MA, United States), respectively. The bacterial 16S rRNA was amplified by using the specific primers 341F (5′-CCTAYGGGRBGCASCAG-3′) and 806R (5′GGACTACNNGGGTATCTAAT-3′) targeting the variable V3–V4 regions. The specific primers ITS5-1737F (5′-GGAAGTAAAAGTCGTAACAAGG-3′) and ITS2-2043R (5′-GCTGCGTTCTTCATCGATGC-3′) were used for fungal library construction by targeting the ITS1 region. Each PCR mixture (50 μl) consisted of 3 μl of DNA template (approximately 60 ng), 25 μl of 2 × SYBR Premix Ex Taq (TaKaRa, Tokyo, Japan), 1 μl of each primer, and 20 μl nuclease-free water. PCR amplification was performed as follows: preincubation at 94°C for 5 min; 30 cycles of 94°C for 30 s, 52°C for 30 s, and 72°C for 30 s; and finally, an extension at 72°C for 10 min. The PCR-amplified products were analyzed by pair-end sequencing using the Illumna MiSeq platform.

The DNA library was constructed following the NEBNext^®^ Ultra^TM^ DNA Library Prep Kit for Illumina^®^ standard procedure (Schloss et al., 2009). The constructed amplicon library was sequenced by PE250 on the Illumina Hiseq2500 platform. The PE reads obtained by MiSeq sequencing were spliced according to the overlap between PE reads, and the sequences were optimized using the software FLASH and Trimmomatic.

Usearch software was used for operational taxonomic unit (OTU) cluster analysis (Edgar, 2010). Bioinformatics statistical analysis of OTUs with 99% sequence similarity was carried out; OTUs were normalized to the same number of reads (27,679 reads in 16S sequence and 59,470 in ITS sequence, the smallest read number of samples) in each sample. Each OTU represented a species (McDonald et al., 2012). Multiple diversity indexes of OTUs were analyzed using the software QIIME (Werner et al., 2012). Based on taxonomic information, a statistical analysis of the community composition was performed at each classification level, and the composition of each sample was counted at each classification level (Price et al., 2010). Bacterial 16S ribosomal DNA (rDNA) gene V3–V4 regions were compared against Greengenes database by using RDP Classifier. Fungal ITS1 regions were compared against Unite database by blast method. Species annotation and abundance analysis were performed using QIIME platform software and KRONA software (Catherine and Rob, 2005). The sequencing data of 16S rDNA have been submitted to the National Center for Biotechnology Information (NCBI) database with accession number PRJNA681733.

### Quantitative Real-Time PCR

The abundance of different groups of microorganisms was determined by quantitative real-time PCR with the whole genomic DNA of microorganisms in each soil sample as a template. The primer sequences and sources are shown in Supplementary Table 1. Each PCR mixture (25 μl) consisted of 1 μl of DNA template (approximately 20 ng), 12.5 μl of 2 × SYBR Premix Ex Taq (Takara, Tokyo, Japan), 1 μl of each primer, and 9.5 μl dd H_2_O. PCR amplification was performed using a Thermal Cycler PCR apparatus (Bio-Rad, Hercules, CA, United States) as follows: preincubation at 94°C for 5 min; 32 cycles of 94°C for 30 s, 54–66°C for 1 min, and 72°C for 1 min; and finally, an extension at 72°C for 10 min. Standard curves were, respectively, generated using serially diluted purified known genomic DNA standard by using the primers presented in Supplementary Table 1. The accuracy of the amplification results was tested by the melting curves.

### Statistical Analysis

The data of soil physicochemical factors and qPCR were analyzed by SPSS (Version 26.0 and IBM Corp., Armonk, NY, United States). Tukey’s honestly significant difference (HSD) test was performed to reveal significant difference. Data were presented as mean ± standard deviation (SD). Origin Pro 2019b was used to draw the box plot of soil physicochemical factors.

## Results

### Control Efficiency

According to Table 1, at 42 days, the incidence of clubroot disease in the control group was 59.8% ± 4.3%, and the disease index was as high as 45.5% ± 2.8%, whereas in the treatment group, it was 21.8% ± 1.6% and 13.9% ± 1.78%, respectively. The control efficiency of *S. alfalfae* XY25^*T*^ on clubroot disease was 69.4%.

**TABLE 1:** Statistics of disease rate, disease index, and control efficiency (day 42).

Group	Control group	Treatment group	*P* value
Disease incidence, %	59.8 ± 4.3	21.8 ± 1.6	<0.001
Disease index, %	45.5 ± 2.8	13.9 ± 1.8	<0.001
Control efficiency, %	–	69.4 ± 2.9	

*Disease incidence (%) = number of diseased plants/total number of counted plants × 100. Disease index (%) = [Σ (number of diseased plants at each stage × relative value)]/(total number of counted plants × highest disease rate) × 100. Control efficiency (%) = (control group disease index - treatment group disease index)/control group disease index × 100.*

### Physicochemical Factors

**FIGURE 1:**
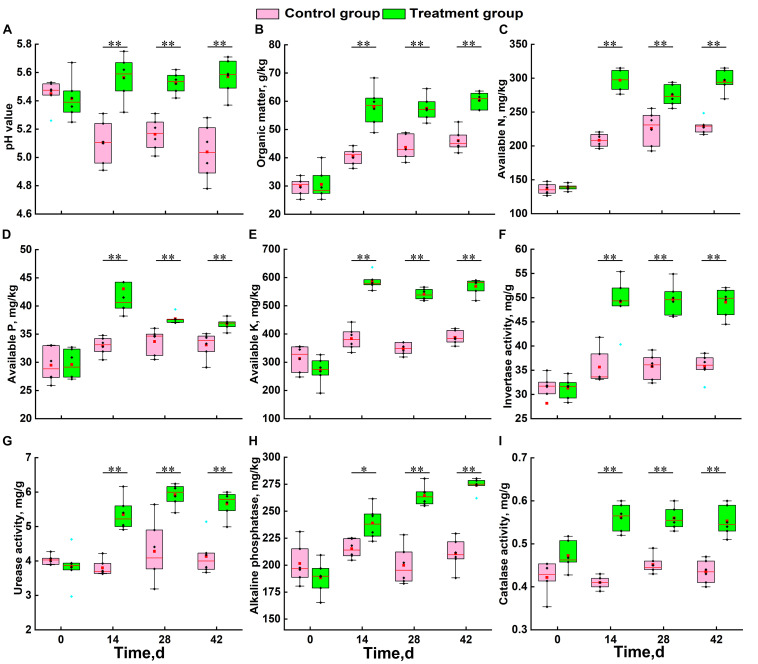
Effect of *S. alfalfae* XY25^*T*^ on physicochemical factors of soil. **(A)** pH value; **(B)** organic matter; **(C)** available nitrogen; **(D)** available phosphorus; **(E)** available potassium; **(F)** invertase; **(G)** urease; **(H)** alkaline phosphatase; **(I)** catalase. *n* = 6; different letters for the same item indicate significant differences at *P* < 0.05 level. The error bars represent the standard deviation (SD). The horizontal bars within boxes represent medians. The tops and bottoms of boxes represent the 75th and 25th percentiles, respectively. The upper and lower whiskers extend to data no more than 1.5 × the interquartile range from the upper edge and lower edge of the box, respectively.

At the beginning of the growing season (day 0), there was no significant difference in physicochemical factors between the two groups (Figure 1). During the growth of Chinese cabbage, the soil pH of the control group continued to decrease, while that of the treatment group showed a slow upward trend. Statistical analysis results showed that the soil acidification trend was significantly alleviated by the application of *S. alfalfae* XY25^*T*^ (Figure 1A). The trends of other physicochemical factors in the treatment group and the control group were basically consistent (Figures 1B–I). The SOM, AN, AP, and AK contents of the treatment group were significantly higher than those of the control group (Figures 1B–E). Compared with the those in the control group, the contents of SOM, AN, AP, and AK in the treatment group at 42 days after transplantation increased by 30.9, 29.0, 11.3, and 50.5%, respectively (Figures 1B–E). The soil enzyme activities of the treatment group were also significantly higher than those of the control group. The soil activities of invertase, urease, alkaline phosphatase, and catalase significantly increased by 37.0, 36.9, 30.1, and 30.4%, respectively.

### Alpha Diversity

High-throughput sequencing was used to analyze the microbial alpha diversity of 24 soil samples (Table 2). The Chao1 index and Shannon index analysis of the bacterial community showed that there was no significant difference in alpha diversity between the two groups. The Shannon index of the control group increased first and then decreased. The change trend of the treatment group was complicated, and it exhibited a lower diversity than the control group. The Chao1 index and Shannon index analysis of the fungal community showed that there were no significant differences in these two indexes between the control groups, but significant differences were observed between the treatment groups. These results showed that the diversity of fungi in the rhizosphere soil was significantly reduced after the application of *S. alfalfae* XY25^*T*^.

**TABLE 2:** Analysis of alpha diversity estimators.

Sample	Bacteria		Fungi	
	Chao1 index	Shannon index	Chao1 index	Shannon index
C0	3,341.8 ± 223.5	8.72 ± 0.39	848.4 ± 5.4*a*	5.05 ± 0.23*ab*
C14	3,295.7 ± 44.5	9.12 ± 0.12	675.9 ± 25.5*abc*	4.19 ± 0.23*abc*
C28	3,080.6 ± 205.4	8.49 ± 0.72	740.5 ± 42.3*ab*	4.47 ± 0.30*abc*
C42	3,274.9 ± 36.5	9.14 ± 0.11	679.2 ± 50.*abc*	4.50 ± 0.69*abc*
S0	3,235.3 ± 64.1	8.94 ± 0.09	796.4 ± 22.8*a*	5.32 ± 0.38*a*
S14	2,769.3 ± 397.3	8.06 ± 0.73	542.2 ± 91.4*bc*	3.41 ± 0.19*c*
S28	2,844.4 ± 379.3	8.41 ± 0.51	472.8 ± 58.2*c*	3.68 ± 0.11*bc*
S42	2,710.2 ± 415.0	8.11 ± 0.82	469.0 ± 55.14*c*	3.31 ± 0.33*c*

**n* = 3; different letters (a, b, or c) for same item indicate significant differences at *P* < 0.05 level; C0, C14, C28, and C42 represent the sampling days 0, 14, 28, and 42 in the control group, respectively; S0, S14, S28, and S42 represent the sampling days of the treatment group.*

### Microbial Community

**FIGURE 2:**
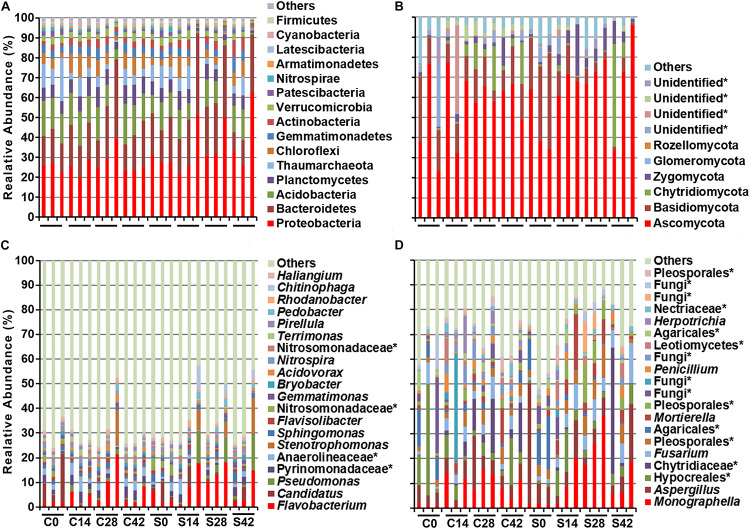
Effect of *S. alfalfae* XY25^*T*^ on microbial community. Bacteria at **(A)** phylum and **(C)** genus level and fungal at **(B)** phylum and **(D)** genus level. C0, C14, C28, and C42 represent the sampling days 0, 14, 28, and 42 in the control group, respectively; S0, S14, S28, and S42 represent the sampling days of the treatment group.

During the whole growth of Chinese cabbage, the bacterial community was composed of 15 phyla, of which Proteobacteria was dominant, followed by Bacteroidetes, Acidobacteria, Planctomycetes, Thaumarchaeota, Chloroflexi, Gemmatimonadetes, Actinobacteria, Verrucomicrobia, Patescibacteria, etc. (Figure 2 and Table 3). A total of six phyla of fungal communities were detected at the phylum level. The most common one was Ascomycota, accounting for 45.7–72.9%, followed by Basidiomycota, Chytridiomycota, Zygomycota, Glomeromycota, Rozellomycota, and four unidentified phylum fungal communities (Figures 2, 4B). In the treatment group, the abundance of Basidiomycota decreased significantly with planting time (Table 4).

**TABLE 3:** Main bacterial groups or the group with significant difference (%).

Group	Control group				Treatment group				*P*-value
	C0	C14	C28	C42	S0	S14	S28	S42	
**At phylum level**									
Proteobacteria	25.3	24.7	31.2	23.9	28.6	32.8	37.4	40.6	0.43
Bacteroidetes	15.3	18.5	26.7	18.2	20.1	27.2	28.9	17.1	0.31
Acidobacteria	16.0	15.5	10.8	16.4	11.9	8.09	9.04	9.95	0.30
Planctomycetes	6.48	7.99	7.14	9.26	6.84	5.99	5.59	4.78	0.42
**At genus level**									
*Flavobacterium*	1.60	4.06	11.4	4.10	3.66	9.00	13.7	6.49	0.19
*Candidatus Nitrosotalea*	9.53	0.62	0.19	0.34	3.73	2.90	0.89	2.13	0.08
*Pseudomonas*	0.29	1.55	1.49	0.92	0.29	4.87	4.69	5.67	0.53
*Stenotrophomonas*	0.02	0.36	5.21	0.35	0.01	3.05	2.46	5.15	0.66
*Sphingomonas*	1.44	1.35	1.42	1.15	2.38	1.48	1.81	2.38	0.55
*Flavisolibacter*	1.33	1.50	1.38	1.48	1.47	1.19	1.00	0.91	0.74
*Gemmatimonas*	1.06	1.35	1.31	1.56	0.90	0.99	0.94	1.34	0.75
*Bryobacter*	1.28	1.22	1.16	1.37	0.92	1.01	1.07	1.23	0.98
*Acidovorax*	0.74b	1.02b	2.02a	1.19ab	0.92b	0.72b	1.42ab	0.86b	0.01
*Pedobacter*	0.31	0.25	1.48	0.40	0.27	1.42	2.23	0.77	0.17
*Chitinophaga*	0.01	0.14	0.13	0.28	0.00	3.23	1.61	1.33	0.60
*Haliangium*	1.06ab	0.56bc	0.49bc	0.63abc	1.22a	0.44bc	0.33c	0.49bc	0.00
*Ferruginibacter*	0.37	1.09	0.71	0.96	0.31	0.65	0.52	0.34	0.05
*Herbaspirillum*	0.18b	0.38b	0.30b	0.18b	0.16b	0.22b	1.12a	0.33b	0.03
*Mizugakiibacter*	0.92a	0.07b	0.22b	0.14b	1.12a	0.07b	0.09b	0.18b	0.00

**n* = 3; different letters (a, b, or c) for same item indicate significant differences at *P* < 0.05 level; C0, C14, C28, and C42 represent the sampling days 0, 14, 28, and 42 in the control group, respectively; S0, S14, S28, and S42 represent the sampling days of the treatment group.*

**FIGURE 3:**
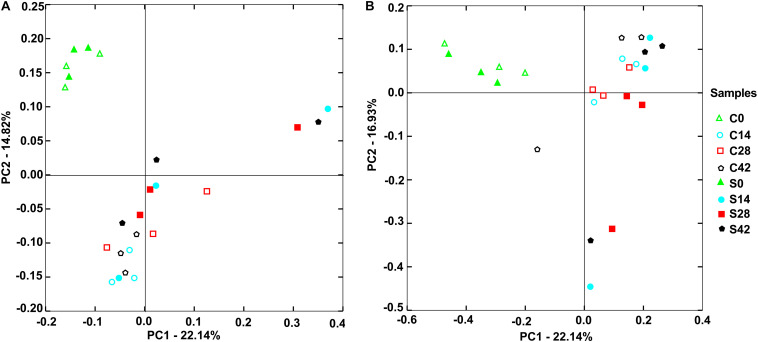
Principal components analysis (PCA) showed distribution of samples. **(A)** Bacterial community and **(B)** fungal community. The closer distance between points shows the higher similarity; *n* = 3.

**FIGURE 4:**
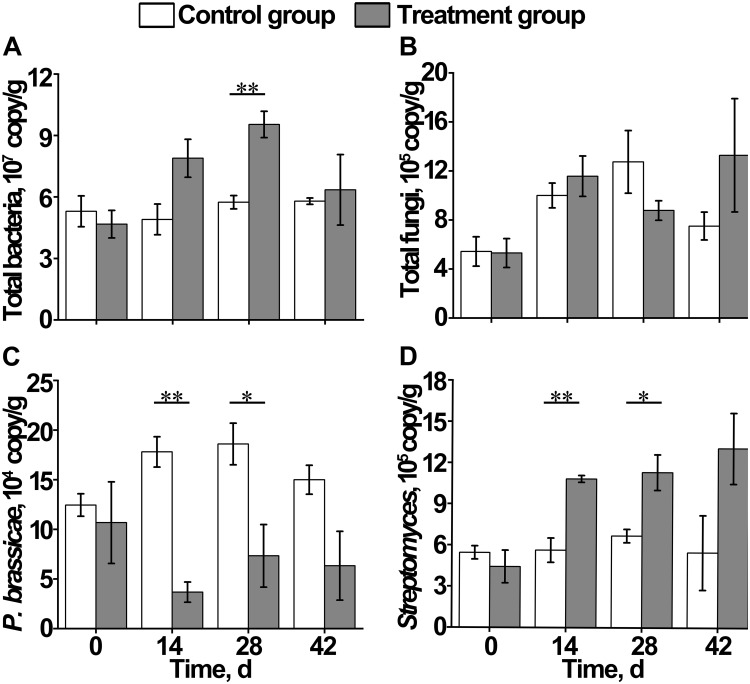
Effect of *S. alfalfae* XY25^*T*^ on content of **(A)** total bacteria, **(B)** total fungi, **(C)** *Plasmodiophora brassicae*, and **(D)** *Streptomyces*. *n* = 3; the letter with * indicates significant differences at *P* < 0.05 level; the letters with ** indicate extremely significant differences at *P* < 0.01 level.

**TABLE 4:** Main fungal groups or the group with significant difference (%).

Group	Control groupy				Treatment group				*P*-value
	C0	C14	C28	C42	S0	S14	S28	S42	
**At phylum level**									
Ascomycota	46.1	59.3	60.3	59.6	45.7	66.8	72.9	67.4	0.57
Basidiomycota	21.8ab	12.1bc	12.2bc	15.6bc	31.1a	9.51bc	5.36c	3.37c	<0.01
Chytridiomycota	0.58	2.95	12.9	8.95	0.88	1.60	0.41	19.6	0.32
Zygomycota	1.17	2.44	2.57	5.72	1.78	12.53	7.51	6.23	0.21
**At genus level**									
*Monographella*	1.28b	9.13b	12.33b	7.78b	1.74b	10.16b	26.98a	10.35b	<0.01
*Aspergillus*	5.70	5.24	10.81	5.69	21.12	8.16	5.40	10.94	0.56
Hypocreales*	19.51	5.54	3.42	8.40	1.52	10.60	2.60	5.72	0.34
*Fusarium*	3.61ab	4.89ab	5.40ab	3.29ab	1.29b	6.38ab	6.50ab	10.36a	0.47
Agaricales-1*	10.07ab	1.50c	1.76c	2.14c	15.96a	0.96c	1.02c	0.32c	<0.01
Chytridiaceae*	0.02	2.69	12.66	8.19	0.01	0.72	0.29	17.37	0.45
Pleosporales-1*	2.12b	2.99b	3.82ab	5.01ab	3.85ab	4.16ab	9.80a	3.49ab	0.04
*Mortierella*	0.94	2.36	2.47	5.58	1.21	11.33	6.43	2.48	0.15
Fungi-1*	11.76	0.56	2.87	1.68	4.42	2.95	0.21	0.00	0.39
Pleosporales-2*	1.17b	2.03b	1.60b	2.40b	1.47b	3.87b	10.43a	2.25b	<0.01
Fungi-2*	0.06b	4.72a	4.33ab	3.16ab	0.06b	1.69ab	0.29b	1.24ab	0.01
*Penicillium*	0.87	3.31	3.05	1.77	1.56	1.85	0.90	3.35	0.48
*Herpotrichia*	0.05	6.64	2.74	1.01	0.02	1.36	0.06	0.36	0.13
*Phaeoacremonium*	0.00	0.00	0.00	0.00	0.00	0.48	0.51	6.62	0.39
Fungi-3*	2.80a	0.32b	0.29b	0.51b	2.19ab	0.54b	2.07ab	0.38b	<0.01
Fungi-4*	0.38b	0.59b	0.51b	1.08b	0.67b	1.00b	6.42a	0.71b	0.04
Agaricales-2*	1.53b	0.09b	0.11b	0.29b	5.37a	0.26b	0.69b	0.17b	<0.01
*Colletotrichum*	0.70a	0.12b	0.10b	0.21b	0.78a	0.06b	0.06b	0.11b	<0.01

*n* = 3; different letters (a, b, or c) for same item indicate significant differences at *P* < 0.05 level; C0, C14, C28, and C42 represent the sampling days 0, 14, 28, and 42 in the control group, respectively; S0, S14, S28, and S42 represent the sampling days of the treatment group. *Next taxon is unknown.

At the genus level, the top 10 bacteria were *Flavobacterium*, *Candidatus*, *Pseudomonas*, *Pyrinomonadaceae*, *Anaerolineaceae*, *Stenotrophomonas*, *Sphingomonas*, *Flavisolibacter*, *Nitrosomonadaceae*, and *Gemmatimonas* (Figures 2, 4C). Statistical analysis showed that there was no significant difference in relative abundance among the top 10 genera. Some minor genera such as *Acidovorax*, *Haliangium*, *Herbaspirillum*, and *Mizugakiibacter* had significant difference in relative abundance. The abundance of *Herbaspirillum* in the treatment group increased significantly at 28 days. The top 20 genera of the fungi are shown in Figures 2, 4D. The top fungi included *Monographella*, *Aspergillus*, *Hypocreales*, *Chytridiaceae*, *Fusarium*, *Pleosporales*, *Agaricales*, *Mortierella*, *Pleosporales*, unidentified fungi, etc. (Figures 2, 4D). Analysis results showed that the most dominant genus, *Monographella*, had significant difference in relative abundance between the two groups. The abundance of *Monographella* in the treatment group was significantly higher than that in the control group at day 28. The significant differences were observed among *Pleosporales*, *Basidiomycota*, *Colletotrichum*, two strains attributed to *Agaricales*, and another two unidentified strains by using *S. alfalfae* XY25^*T*^.

### Principal Component Analysis

As shown in Figure 3A, for bacterial communities, the horizontal axis PCA1 accounted for 22.14%, and the vertical axis PCA2 occupied 14.82%. At the beginning (day 0), the bacterial community composition in the control group (C0) was similar to that in the treatment group (S0). With the growth of Chinese cabbage, the bacterial community composition in the rhizosphere soil changed, and the bacterial community compositions on days 14, 28, and 42 were far away from that at day 0. In the control group, the community compositions of the three replicates were similar. Compared with that in the control group, the bacterial community composition in the treatment group exhibited greater change, and the three replicates of treatment were farther apart (Figure 3A).

For the fungal community, the horizontal axis PCA1 accounted for 22.14%, and the vertical axis PCA2 occupied 16.93% (Figure 3B). The control group C0 and the treatment group S0 had similar fungal community composition. With the extension of growth time of Chinese cabbage at days 14, 28, and 42, there was a large difference between two groups. Compared with those in the control group, the difference among the three replicates in the treatment group was large (Figure 3B). These results indicated that the application of *S. alfalfae* XY25^*T*^ affected the microbial community in rhizosphere soil.

### Correlations Between Microbial Community and Soil Edaphic Factors

As shown in Supplementary Figure 1A, the main bacterial communities that were significantly negatively correlated with soil pH were Ferruginibacter, Ellin6067, and UTCFX1. *Flavobacterium* was extremely positively correlated with AN, and this community was significantly positively correlated with urease, catalase, AP, and alkaline phosphatase. *Pseudomonas* and *Chitinophaga* were significantly positively correlated with SOM, AN, AP, AK, invertase, alkaline phosphatase, and urease. *Stenotrophomonas* was significantly positively correlated with SOM, AP, invertase, AN, and phosphatase. *Flavisolibacter* was significantly negatively correlated with urease and AN. *Pedobacter* was significantly positively correlated with urease, AN, and alkaline phosphatase. Other major communities such as *Candidatus*, *Nitrosotalea*, *Sphingomonas*, *Gemmatimonas*, *Bryobacter*, and *Acidovorax* exhibited no significant relationship with each factor.

As for fungi, soil pH was extremely significantly positively correlated with *Junewangia*, *Rhizopus*, and *Phaeoacremonium*; extremely negatively correlated with *Leptosphaeria*, *Aphanoascus*, and *Robillarda*; and significantly negatively correlated with *Penicillium*. *Monographella* was negatively correlated with SOM, AN, invertase, and alkaline phosphatase and negatively correlated with soil AP, AK, urease, and catalase. *Aspergillus* was extremely positively correlated with AK and positively correlated with alkaline phosphatase, AP, and SOM. *Fusarium* displayed a very significant positive correlation with alkaline phosphatase. *Mortierella* was significantly positively correlated with invertase and SOM and significantly positively correlated with AN and AK (Supplementary Figure 1B).

### Microbial Population Determination Through Quantitative Real-Time PCR

During the growth of Chinese cabbage, the total bacterial content in the soil of the control group remained basically stable, whereas that of the treatment group increased first and then decreased, which was significantly higher on day 28 than on day 0 (Figure 4A). The total fungal content in the soil of the control group and the treatment group both increased first, and then, the control group reached the maximum value on day 28, followed by a decrease, whereas the treatment group reached the maximum value on day 42. However, no significant differences were observed between the control group and treatment group at each time point (Figure 4B). On day 14, the content of *Plasmodiophora brassicae* in the control group was significantly higher than that in the treatment group, indicating that there were signs of clubroot disease occurrence in Chinese cabbage on day 14 (Figure 4C). The content of *Streptomyces* in the control group remained stable, and its content in the treatment group gradually increased, which was significantly higher than that in the control group on day 42 after transplantation (Figure 4D).

## Discussion

Chinese cabbage is often threatened by clubroot disease in its growth process. Generally, the clubroot disease occurs about at 30 days after the Chinese cabbage transplantation and eventually breaks out in an all-round way, resulting in crop yield reduction by more than 80% in severe cases (He et al., 2019). Biological methods have potential application prospects in the prevention and treatment of clubroot diseases. *Lactobacillus plantarum* PM411 and *Lactobacillus plantarum* TC92 can control the occurrence of plant diseases under relatively suitable humidity (Núria et al., 2018). One previous study has reported that *Streptomyces platensis* 3–10 inhibits the growth of spores of *P. brassicae in vitro* (Shakeel et al., 2016). The greenhouse experiment results have showed that the strains of endophytic actinomycetes can inhibit the occurrence of clubroot disease of Chinese cabbage (Lee et al., 2008; Liu et al., 2020). Based on the control efficiency of *S. alfalfae* XY25^*T*^ obtained in the greenhouse experiment, the field experiment was further carried out and showed that the clubroot disease incidence of Chinese cabbage was significantly reduced by applying *S. alfalfae* XY25^*T*^, and the control effect was 69.4%. We hypothesized that the control efficiency of *S. alfalfae* XY25^*T*^ might be associated with changes in rhizosphere microbial community. Therefore, the rhizosphere samples of Chinese cabbage were collected to study the composition of microbial community by high-throughput sequencing and real-time PCR.

Microbial communities play an important role in soil structure and disease control of plants (Xue et al., 2018). In recent years, high-throughput sequencing technology has deepened the study of the diversity of soil microbial communities (Roesch et al., 2007; Blaalid et al., 2012; Liu et al., 2018). In this study, high-throughput sequencing and qPCR were used to study the microbial community during the occurrence of Chinese cabbage clubroot disease. The results showed that at the phylum level, Proteobacteria was the most dominant bacteria with an abundance of 23.9–40.6%, followed by Bacteroidetes, Acidobacteria, and Plantomytomytes. Acidobacteria has been reported to be one of the five largest taxa in the soil (Roesch et al., 2007; Liu et al., 2018), and previous studies have shown that Proteobacteria is the most dominant bacteria in the soil (Delgado-Baquerizo et al., 2018), followed by Basidiomycota (Chowdhury et al., 2013; Liu et al., 2018). Planctomycetes is also one of the main communities in this study, and this result is different from many other findings (Roesch et al., 2007). In this study, the largest bacterial group at the genus level was *Flavobacterium*, whose abundance in the treatment group was about 2% higher than that in the control group, but there was no significant difference between these two groups. *Flavobacterium hercynium* EPB-C313 has been found to have a significant inhibitory effect on *P. brassicae* (Hahm et al., 2012). A previous study has shown that *Bacillus subtilis* XF-1 can promote the growth of *Flavobacterium* and that *Flavobacterium* has a potential role in the prevention and treatment of clubroot disease (Liu et al., 2018). Our study indicated that the major microbial groups were “*Candidatus* Nitrosotalea,” *Pseudomonas*, *Stenotrophomonas*, *Sphingomonas*, *Flavisolibacter*, and *Gemmatimonas* in terms of abundance order. “*Candidatus* Nitrosotalea” is a representative of ammonia-oxidizing archaea, which is common in soil, and it plays an important role in the nitrogen cycle (Lehtovirta-Morley et al., 2016). *Pseudomonas* and *Stenotrophomonas* are common taxa in soil (Dai et al., 2020). Our data showed that after applying *S. alfalfae* XY25^*T*^, the top 10 most abundant genera did not change significantly, but a few genera showed significant changes. For example, at 28 days after transplantation, the abundance of *Herbaspirillum* in the treatment group increased significantly. *Herbaspirillum* plays an important role in the circulation of nitrogen to promote the growth of sugarcane (Manoel da Silva et al., 2015).

In this study, the most dominant fungus at the phylum level was Ascomycota with its abundance ranging from 45.7 to 72.9%, which was consistent with a previous study finding that Ascomycota was the most abundant phylum in the soil, reaching 29.98–45.69% (Liu et al., 2018). Our data showed that the second dominant fungus was Basidiomycota. Interestingly, the abundance of Basidiomycota in the treatment group was 31.1% at day 0, but it was only 3.37% at day 42, indicating that the application of *S. alfalfae* XY25^*T*^ significantly decreased its Basidiomycota abundance as the crop grew. Our result was similar to a previous study report that Basidiomycota was the second-ranked phylum whose abundance was much lower than Ascomycota (Liu et al., 2018). However, some studies have reported different findings that the main phylum in rhizosphere soil was Basidiomycota (Buee et al., 2009; Blaalid et al., 2012). Our results indicated that at the genus level, the most abundant fungi were *Monographella*, *Aspergillus*, *Hypocreales*, *Chytridiaceae*, *Fusarium*, *Pleosporales*, *Agaricales*, *Mortierella*, and *Pleosporales*, which was not completely consistent with a previous study reporting that *Fusarium*, *Cryptococcus*, and *Mortierella* were the most abundant fungi (Liu et al., 2018). Our data indicated that the application effect of streptomyces on soil fungal communities is different from that on bacterial communities and that the abundance of many fungal communities changed significantly at different time points. For example, the abundance of *Monographella* was significantly higher in the treatment group than in the control group at 28 days. There were significant differences in abundance among other fungal communities belonging to *Agaricales*, *Pleosporales*, *Basidiomycota*, *Colletotrichum*, and two unknown fungi. There are many nonculturable microorganisms in the soil, of which these nonculturable fungi play an important role in the formation of clubroot disease. This study showed the application of *S. alfalfae* XY25^*T*^ had a great influence on the fungal community, which might be one of the reasons for preventing and controlling clubroot disease by this strain.

The imbalance of rhizosphere microbial community composition is an important cause of soil-borne diseases. Soil biodiversity and soil microbial composition determine ecosystem multifunctionality and soil bacterial diversity, which exert an inhibitory effect on the plant pathogen (van Elsas et al., 2012; Cameron et al., 2014). Generally, the richer the microbial community composition, the stronger the ability to inhibit pathogens (Raaijmakers et al., 2009). This study showed that during the growth of crops, soil bacterial diversity decreased first, and then increased, and fungal diversity significantly decreased after applying *S. alfalfae* XY25^*T*^. PCA analysis results showed that the application of *S. alfalfae* XY25^*T*^ affected the microbial communities in the rhizosphere soil. Our qPCR results showed that the contents of total bacteria and total fungi remained unchanged or increased slightly. These results indicated that application of *S. alfalfae* XY25^*T*^ could cause some types of microorganisms to aggregate and grow. Moreover, qPCR results also showed that the content of *P. brassicae* in the treatment group was significantly higher than that in the control group on day 14 after transplantation. On day 28, clubroots were observed. The above results suggested that the application of *S. alfalfae* XY25^*T*^ reduced the incidence of clubroot disease of Chinese cabbage by inhibiting the growth of *P. brassicae*.

Soil microorganisms could effectively reflect soil fertility. Soil acidity, fertility, and enzyme activity are closely related to the activities of microorganisms, and these factors jointly affect the composition of microorganisms (Zhang et al., 2016; Rui et al., 2017; Xue et al., 2018). In general, the content of microorganisms is directly proportional to soil fertility. This study showed that the application of *S. alfalfae* XY25^*T*^ could significantly alleviate the trend of soil acidification and improve soil fertility and enzyme activity. Changes in soil enzyme activity have an important impact on the formation of soil organic matter and nutrient cycle (Liu et al., 2013). The application of microbial agents promotes the growth of microorganisms and enhances their activities so that more enzymes are produced, which is conducive to the production of organic matter.

## Conclusion

In summary, *S. alfalfae* XY25^*T*^ could be used to reduce the clubroot incidence on Chinese cabbage in the outbreak area. After applying *S. alfalfae* XY25^*T*^, the community of rhizosphere microorganisms in Chinese cabbage changed, and the fungal diversity decreased significantly. Furthermore, the application of *S. alfalfae* XY25^*T*^ also improved the soil fertility, reduced soil acidification, and inhibited the growth of *P. brassicae*. This study provides a better understanding of the importance of the microbial community in soil clubroot disease, and it also offers an effective strategy and theoretical basis for biological control of clubroot disease.

## Data Availability Statement

The datasets presented in this study can be found in online repositories. The names of the repository/repositories and accession number(s) can be found below: https://www.ncbi.nlm.nih.gov/, PRJNA681733.

## Author Contributions

YL, SZ, and ZqZ designed the study. LQ and KL performed the experiments. YlH and YmH drafted the manuscript. ZjZ, XX, and SX analyzed the data. YlH and ZjZ contributed to the manuscript revision. ZqZ, SX, and SZ were the responsible for the visualization, supervision, project administration, and funding acquisition. YmH and YL contributed to the overall support of this study. All authors read and approved the final manuscript.

## Conflict of Interest

The authors declare that the research was conducted in the absence of any commercial or financial relationships that could be construed as a potential conflict of interest.

## Supplementary Material

Supplementary Figure 1 Correlations between soil physicochemical factors and genus of microbial community.

Supplementary Table 1 Primers used for quantitative real-time PCR.
