# Supplementary material for: Control of Streptomyces alfalfae XY25T Over Clubroot Disease and Its Effect on Rhizosphere Microbial Community in Chinese Cabbage Field Trials
Source: Front Microbiol. 2021 Jun 18;12:641556. doi: 10.3389/fmicb.2021.641556 (PMC8253263; doi:10.3389/fmicb.2021.641556)
Supplement: Supplementary Figure 1 — Correlations between soil physicochemical factors and genus of microbial community. [file Data_Sheet_1.docx]

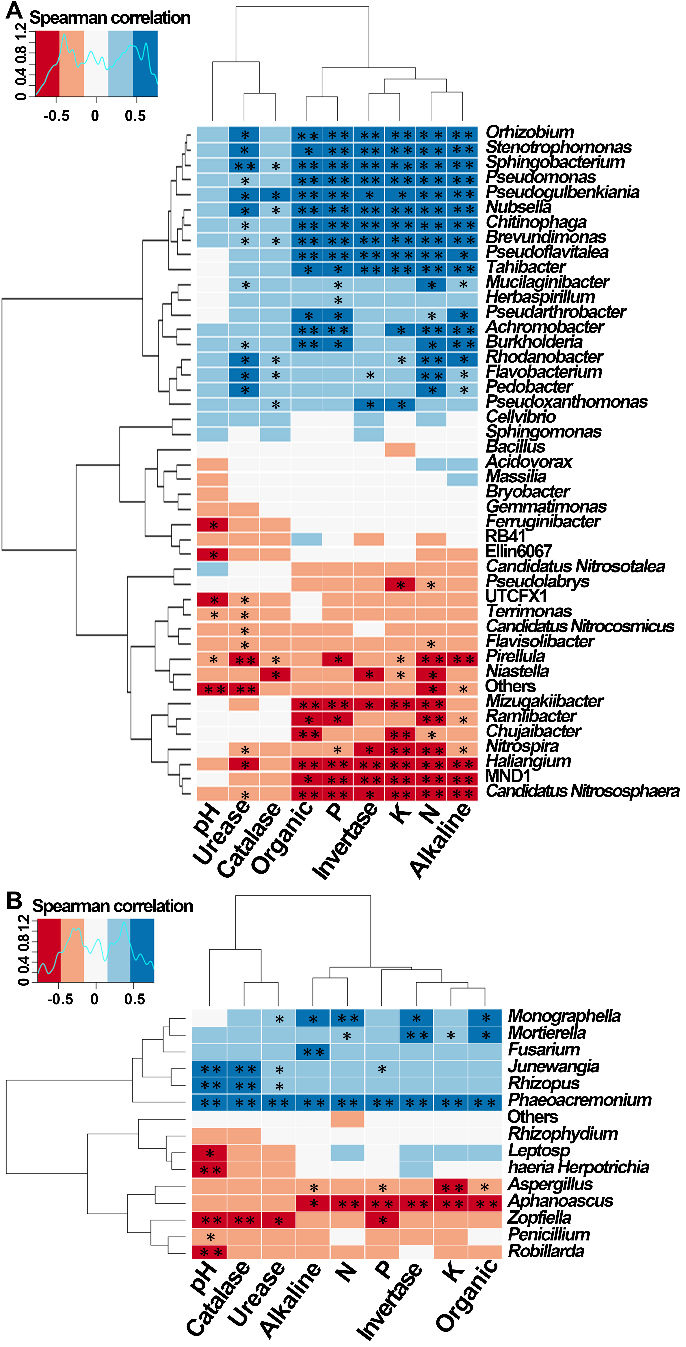


**Figure S1. Correlations between soil physicochemical factors and** [**genus**](file:///C:\Administrator\AppData\Local\youdao\dict\Application\8.1.2.0\resultui\html\index.html#/javascript:;) **of microbial community.** Main bacterial genera(A); Main fungal genera (B); The letter of * indicates significant differences at *P* < 0.05 level, the letters of ** indicates extremely significant differences at *P* < 0.01 level.

**Table S1. Primers used for quantitative real-time PCR**

| **Group** | **Prime Sequence (5′→3′)** | **Amplicon size** | **Annealing temp** | **Reference** |
| --- | --- | --- | --- | --- |
| Total bateria | GTGCCAGCMGCCGCGGTAA (515F)  GGACTACHVGGGTWTCTAAT (806R) | 299 bp | 56 °C | (Carini et al., 2016) |
| Total fungi | GCATCGATGAAGAACGCAGC (ITS3F)  TCCTCCGCTTATTGATATGC (ITS4R) | 400 bp | 54 °C | (Kurzemann et al., 2020) |
| *Plasmodiophora brassicae* | AAACAACGAGTCAGCTTGAATGC (PbF)  TTCGCGCACAAGCACTTG (PbR) | 104 bp | 59 °C | (Wallenhammar et al., 2012) |
| *Streptomyces* | GAGTACATCGAGATGCGCCGCAA (AMgeoF)  GAGAAGAGGTCGTT-GCGCAGGTG (AMgeoR) | 104 bp | 66 °C | (Auffret et al., 2011) |

**References**

[1] Auffret, M., Pilote, A., Proulx, É., Proulx, D., Vandenberg, G., and Villemur, R. (2011). Establishment of a real-time PCR method for quantification of geosmin-producing Streptomyces spp. in recirculating aquaculture systems. *Water Res.* 45**,** 6753-6762.

[2] Carini, P., Marsden, P.J., Leff, J.W., Morgan, E.E., Strickland, M.S., and Fierer, N. (2016). Relic DNA is abundant in soil and obscures estimates of soil microbial diversity. *Nat. Microbiol.* 2**,** 1-6.

[3] Kurzemann, F.R., Plieger, U., Probst, M., Spiegel, H., Sandén, T., Ros, M., and Insam, H. (2020). Long-term fertilization affects soil microbiota, improves yield and benefits soil. *Agronomy* 10**,** 1664.

[4] Wallenhammar, A.C., Almquist, C., Söderström, M., and Jonsson, A. (2012). In‐field distribution of Plasmodiophora brassicae measured using quantitative real‐time PCR. *Plant Pathol.* 61**,** 16-28.
